# Balancing influence between actors in healthcare decision making

**DOI:** 10.1186/1472-6963-11-85

**Published:** 2011-04-19

**Authors:** Robert M Kaplan, Yair M Babad

**Affiliations:** 1 Departments of Health Services and Medicine, University of California, Los Angeles, USA; 2 Information and Decision Sciences, College of Business Administration and Liautaud Graduate School of Business, University of Illinois at Chicago, Chicago, USA

## Abstract

**Background:**

Healthcare costs in most developed countries are not clearly linked to better patient and public health outcomes, but are rather associated with service delivery orientation. In the U.S. this has resulted in large variation in healthcare availability and use, increased cost, reduced employer participation in health insurance programs, and reduced overall population health outcomes. Recent U.S. healthcare reform legislation addresses only some of these issues. Other countries face similar healthcare issues.

**Discussion:**

A major goal of healthcare is to enhance patient health outcomes. This objective is not realized in many countries because incentives and structures are currently not aligned for maximizing population health. The misalignment occurs because of the competing interests between "actors" in healthcare. In a simplified model these are individuals motivated to enhance their own health; enterprises (including a mix of nonprofit, for profit and government providers, payers, and suppliers, etc.) motivated by profit, political, organizational and other forces; and government which often acts in the conflicting roles of a healthcare payer and provider in addition to its role as the representative and protector of the people. An imbalance exists between the actors, due to the resources and information control of the enterprise and government actors relative to the individual and the public. Failure to use effective preventive interventions is perhaps the best example of the misalignment of incentives. We consider the current Pareto efficient balance between the actors in relation to the Pareto frontier, and show that a significant change in the healthcare market requires major changes in the utilities of the enterprise and government actors.

**Summary:**

A variety of actions are necessary for maximizing population health within the constraints of available resources and the current balance between the actors. These actions include improved transparency of all aspects of medical decision making, greater involvement of patients in shared medical decision making, greater oversight of guideline development and coverage decisions, limitations on direct to consumer advertising, and the need for an enhanced role of the government as the public advocate.

## Background

The product of the healthcare system should be the health of the population served by the system[1] even though other objectives such as fairness, equity and responsiveness are also important considerations in the allocation of healthcare resources. Yet, the impact of healthcare on health is rarely measured, discussed, or effectively used for resource allocation. One purpose of this paper is to argue that services and resources in healthcare should be directed toward maximizing the public health. We use the United States as an example because of its extreme consumption of healthcare resources, and because it is currently implementing a healthcare reform. Still, many if not all of the considerations herein are also applicable to other healthcare systems.

**Figure 1:**
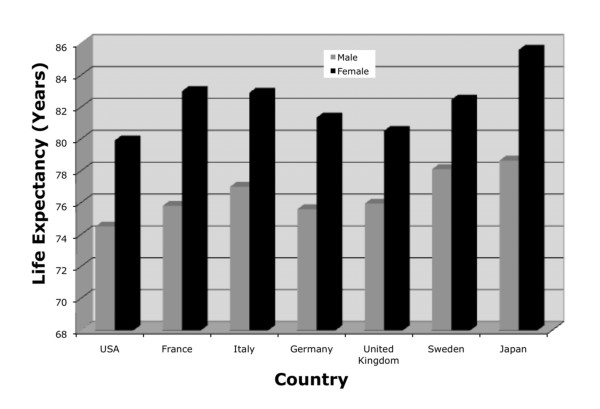
Life expectancy in selected developed countries, 2005 (OEDC data, art original)

Several major issues, which are most evident in the United State (U.S.), must be addressed with regard to healthcare resources and their allocation. First, health outcomes in the U.S. are not outstanding relative to other developed economies, even though the U.S. healthcare expenditure per capita and as a percentage of GDP far exceed those of any other economies[2,3]. Among G8 countries, the U.S. has higher infant mortality and lower life expectancies than peer societies (Figure 1). Second, the U.S. has been unable to control healthcare costs. Although the rate of increase slowed down in the 1990s[4], evidence suggests a new acceleration in healthcare costs and that costs will continue to increase in the future[5]. The consequences of the inability to control costs are well known, and are among the major drivers for the current health reform. For example, American products do not compete well on the international market[6], and the rate of uninsured which appears to be linked to the costs of healthcare[7] continues to climb. Third, the distribution of health services and outcomes is highly variable, with wide differences in their distribution among various population groups.

In March, 2010, U.S. President Obama signed the "Patient Protection and Affordable Care Act" (P.L.111-148). This complex set of regulations will be phased in over several years. The first phase began in September 2010. Insurance companies can no longer deny coverage to children who have preexisting medical conditions and insurance companies are required to provide coverage on the parents policies for young adults up to age 26. The first phase also included creation of a temporary high-risk pool for those with pre-existing conditions. By 2014 most U.S. Citizens and qualified legal residents will be required to have health insurance. Large employers will be required to offer health insurance, or provide their employees with a tax credit for purchasing health insurance. In order to give all citizens the benefits of being under the wings of a large healthcare purchaser, the states will create health insurance exchanges that are accessible to individuals and small groups.

While the objective of healthcare is to produce a healthy population, much of the current focus is on the wrong metrics. Health outcome can be defined as a product of life expectancy and quality of life, and there is wide agreement that healthcare systems should maximize average healthy life expectancy[8]. But, instead of measuring health outcomes, healthcare systems often measure variables that may not directly affect health outcome. Productivity has been measured by counting units of services delivered, and quality has been defined by number of units of service that comply with guidelines. As a result, many resources are used to support services that have little impact on patient outcomes, while other services that may have a substantial impact on health outcomes are underutilized. The problem, we argue, stems from the imbalance between the resources, information, and motivation of the various healthcare arena actors. In this paper, we highlight these differences through the presentation of a simplified model that includes only three categories of actors: individuals, enterprises, and government. We also suggest policies that might provide greater balance between the actors, and help improve population health.

### The Misalignment of Incentives

Healthcare resource allocation should focus on the objective of providing the most health, rather than the most services, to most people. This is a society-wide objective, and not an individual-oriented or enterprise-oriented goal. Rather than providing the most services to many individuals, healthcare systems should strive to raise the health status of the population to its highest possible level.

In the preamble to its 1946 Constitution the WHO's defined health as "A state of complete physical, mental and social well-being." The Ottawa Charter of 1986 expanded the definition to include health as a resource for living, including social and personal activities in addition to physical functioning. But buying health and buying healthcare are not equivalent, and health outcomes may not be achieved by service delivery alone. Most developed counties face a paradox of healthcare excess and deprivation. Some people get too much healthcare, including expensive but unnecessary or ineffective tests and treatments[9], while others get too little or insufficient care, or none at all. Experts believe that in the U.S., which is perhaps the most extreme example, between one-third and one-half of all of the services purchased and delivered have no beneficial effect on individual health outcomes[10].

Overuse of services that do not result in better patient outcomes can occur when incentives and structures are not aligned with achieving the objective of improved population health. The U.S. healthcare market is an example of a system that incentivizes use over outcome. Overuse and misuse of services has negative consequences for several reasons. First, excessive use increases the costs of healthcare. Second, overuse of ineffective health services such as treatments, tests and medications used for purposes not directly linked to improving health contributes to the increase in healthcare costs. Third, certain segments of the population are offered healthcare coverage matching their ability to pay but far exceeding their direct needs, and the cost of the additional services further increases the costs of the health services for the whole population.

In a kind of paradox, excess and deprivation are causally linked. Over- and inappropriate consumption cause health services costs and insurance rates to rise, leading to an increase in deprivation for other, and particularly the most vulnerable, segments of the population. This is best illustrated in an analysis of the U.S. system reported by Gilmer and Kronick[11] who demonstrated that between 1979 and 2002, inflation adjusted per capita health expenditure and the percentage of workers who are uninsured track almost perfectly. Increases in the cost of health are very highly correlated with the percentage of the workforce that is uninsured. In the U.S. healthcare system, many of the increased costs are passed on to employers who still pay most of the health insurance costs, as well as to public healthcare agencies (e.g., Medicaid, Medicare) and to individual health policy holders. As costs increase, many employers decide to discontinue coverage for their employees, and other insureds drop their coverage. The result is an increase in the uninsured rates, as well as an increase in the percentage of those electing to have minimal or insufficient health coverage, with an overall increase in the deprivation of health coverage[12,13]. Today, nearly 50 million U.S. citizens are without health insurance[14] and many others have minimal coverage or are only covered for catastrophic events. In other countries with universal coverage, excessive consumption may not influence the number of people left out of healthcare system; still, overconsumption strains the system, resulting in the elimination or minimization of other services, poorer working conditions for providers, and increased taxes.

Another link between excess and deprivation is reflected in the development of health outcomes over time. Excess treatments for working age individuals indeed reduce - for those receiving these - the incidences of mortality and severe morbidity[15]. The result is an increase in the number of older living adults who, due to their limited resources and the lack of continued health insurance from employers, are eventually deprived of healthcare relative to their working periods, even if they receive public medical services.

### Health Outcomes and Health-Related Quality of Life

Metrics are needed to measure and assess the performance of the healthcare system in improving the population health. This should be done on two levels. First, the improvement should be measurable at the level of the individual. Second, it must conceptualize and measure health status on the larger scale of population and society. The conceptualization and measurement of health has been a concern of scholars for many decades[16]. Public-health statistics concentrate on mortality and death rates. One major health indicator is life expectancy, defined as the expected number of years of life for a member of the considered population. A second major indicator is infant mortality, defined as the number of babies born alive that die within one year of birth, and is usually expressed per 1,000 live births. Mortality remains the major outcome measure in most epidemiological studies and clinical trials, and many public-health statistics focus exclusively on mortality, due to the ease of measurement of crude mortality rates, age-adjusted mortality rates, and infant mortality rates.

Obviously, any model of health outcome that excludes mortality would be incomplete. However, many significant health conditions are not well reflected by mortality information, even though they affect function and quality of life. For example, osteoarthritis, cataracts, and minor depression may all cause poor health with little or no effect on life expectancy or infant mortality statistics.

The measurement of health must consider not only a person's current ability to function, but also the probability of future changes in function, in addition to the probability of death. The spectrum of medical care ranges from public health, preventive medicine, and environmental control, to diagnosis, therapeutic intervention, convalescence, and rehabilitation. Many of these affect the probability of future dysfunction, rather than or in addition to altering present functional status. In many aspects of preventive care, for example, the benefits of the treatment are evident only many years after the intervention. A supportive family that instills proper health habits in its children also promotes better health in the future, even though the benefit may not be realized for years. A person who is very functional and asymptomatic today may harbor a disease with a poor prognosis. For example, many individuals are at high risk of dying from heart disease even though they are perfectly functional today. Should we call them "healthy?" Comprehensive models that combine morbidity, mortality, and prognosis have been described in the literature[17], resulting in health outcome measures that combines life expectancy with quality of life and functioning. We prefer the concept of the quality-adjusted life years (QALY), which offers a general summary of population health outcome. The primary advantages of the QALY are that it is generic, and that it can be used as the measurable target of health maximization. The goal of optimizing health for a population can be achieved by maximizing QALYs within the constraints of the resources, as it can also be achieved by other measures^1^.

We recognize that different players have different motivations for participating in health care. From our perspective, the key to enhancing health is to focus on maximizing health outcomes, rather than on expanding units of delivered service [18], or investing almost exclusively in medical care. Some services enhance health outcomes, while others have little effect. Optimizing population health may best be achieved by investing in the combinations of activities that maximize health outcomes. In later sections, we will talk about strategies to improve outcome. But before we do, it is important to understand the healthcare systems to be optimized, and the imbalances that led to may of the current problems and to the health opportunity cost dilemma.

### The Healthcare Arena and Actors

**Figure 2:**
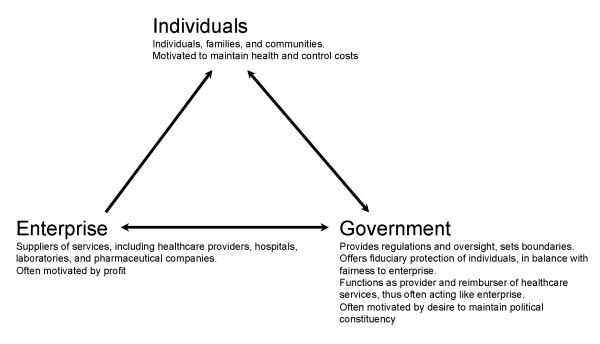
The three actors in health. Note two way arrows between enterprise and government and government and individuals, but one way arrow between enterprise and individuals (art original)

To better understand the essentials of the healthcare arena, and not be mired by its many complexities and details, we consider how healthcare usage and financing decisions are made and implemented in an extremely simplified modeled environment. While many sociological, political and organizational processes affect the healthcare arena, we concentrate here on basic economic processes which shape this environment. We simplify the discussion by focusing on three major groups of participants, or "actors", that affect the intricate healthcare arena (see Figure 2). There are many players within each group, and they are often competing with each other. Further, often participants may participate in several groups. Still, the similarities in interests, objectives and resources among members of the same group exceed the differences between the groups.

At the "receiving end" is the ***individual ***actor; this is the current or future patient who does or will receive health-related services, treatments and medications from the healthcare system - the ultimate consumer of the system's services. Actually, this actor may be either a single individual such as a patient seeing a physician, a group of individuals like a family, or a community or the public in the case of epidemics. The individual consumer is the final determinant of the healthcare scope and services, which eventually have to appease and fulfill his or her, and the myriad of other individuals, expectations. The individual's goal is to maintain or improve his health status and outcomes, at a "reasonable" cost [19,20]

The individual is the eventual payer for the healthcare system services, in one or more ways: direct payout for services; a (direct) health tax; through allocations from public sources that are eventually financed by taxes' paid directly by individuals or indirectly by other participants in the market; or through premiums paid by individuals or by employers in lieu of increased salaries. For example, it is naïve to suggest that getting more benefits paid by insurance companies reduces costs. When companies are mandated to pay for a particular service, these extra costs are usually reflected in higher insurance premiums or in limitations of other services. Employees who get richer health insurance packages might unknowingly be accepting lower wages. As noted by Fuchs [21], many maneuvers described as lowering health care costs, are actually cost shifts that do not reduce costs.

At the "supplier end" is the ***enterprise ***actor, who provides the various services to the individual. That actor can be a direct supplier of health related services, such as a medical provider of ambulatory or emergency health services, a short stay hospital, or a long-term nursing home. The enterprise can be a support and supply entity, such as a lab, a drug company, a distributing drug store, or a provider of preventive services and education. The enterprise can be a financing or management entity, which caters to the healthcare arena, such as a HMO, an insurance company, or another public or private payer. An enterprise might also be an investor in goods that affect health outcomes, such as education or public facilities. The enterprise is often profit oriented, though not for profit organizations are also included. Enterprise decisions are most often influenced by economic factors, but political, organizational, knowledge, and other forces can also motivate enterprise actions.

The third actor is the ***government***, the representative of the people, whose goal should be to use available public and private resources effectively and efficiently to maximize population health status and outcomes. The government is the policy maker and legislator that sets the boundaries and playing field for the individuals and enterprises; in this role it often strives to satisfy the expectations of individuals and maintain fairness toward and between enterprises, while also generating new expectations. A non-government health authority could also play this role. The government (or substitute health authority) also fulfills the role of controller and auditor which assures that the other actors "play according to the rules". It licenses providers, sets the boundaries for their activities and audits them to assure that they follow the legislations and rules. As the public advocate, the government is best suited to provide a long-range vision of public health and healthcare systems. It has the power to direct and monitor population health, and evaluate and execute - directly or indirectly - a wide range of activities that might enhance population health status.

On the other hand, the government has two other roles closely related to the enterprise. It may have a direct role as an enterprise, either as a direct provider of healthcare services, or as a reimburser and payer for medical services. In the U.S., the government owned VA Health Systems is a direct provider of services, and the Centers for Medicare and Medicaid Services (CMS), which operates plans like Medicare and Medicaid, reimburses providers for healthcare services. In this role CMS determines the scope of services provided, and affects the level of healthcare services through the encouragement and discouragement of particular service through regulations and direct payments to providers. Other government agencies, like the Securities and Exchange Commission (SEC), promote and encourage the profit orientation of the private enterprises, regardless of their impact and contribution to the population health. Consequently, the government is often in a conflict of interests between its various roles.

The same model applies to many countries, and similar actors exist everywhere. The Ministry of Health in Israel offers one example. This agency is responsible for the implementation of the National Health Act and for the regulation of the national health basket of treatments and medications, and at the same time it is the operator of about half the public hospitals. Other international examples are available [22]. At the same time, the existence of a national health system tends to emphasize the role of government. Where national health systems do not exist, or where market emphasis is evident as in the U.S., the enterprise has significant power and role in the healthcare arena.

A significant driver of the healthcare environment and health outcomes is the availability and control of health related information, and the inherent balance between the actors. The actions of individuals, as well as their perception of a desirable health status and outcomes, are driven by their expectations for health outcomes. Due to lack of knowledge, these are often substituted with expectations for health services, which are assumed to be surrogates for health outcomes. These expectations are often set by the enterprise or the government, either directly through related agencies or by the political process. Government and enterprise have the information, knowledge, aura of expertise, and resources to gain attention for these issues. The individual and the public function from a position of weakness, often with minimal or partial information. The public may have high expectations, which are significantly affected by the enterprise and government, but the process of their fulfillment - and the resulting shaping of the healthcare system - may be warped and convoluted.

### The Healthcare Market Imbalance

This "triangle of actors" is, to a large extent, at the source of the opportunity cost dilemma which is reflected by the paradox of excess and deprivation. The key problem is the lack of efficiency of the healthcare market, which is driven to a large extent by the imbalance of information and power between the actors. In an efficient market the prices of services and assets reflect information known to all the participants, are unbiased and embody the collective beliefs about future prospects. It is not possible to consistently outperform an efficient market. But in the healthcare market the individual has the least resources and has the least knowledge, either due to the highly specialized and complex nature of the medical and health information and lack of education, or due to lack of access to sources of information. The lack of sufficient resources and information further hampers the individual, especially at times of need, since he often lacks the ability to personally withstand the lengthy and costly treatments faced in a time of crisis.

The enterprise and the government, on the other hand, hold most of the cards. They have the information and expertise to understand and interpret it, and the resources to use the information. Many enterprises have vast resources, and especially the pharmaceutical companies, insurers, and large healthcare organizations; this is particularly evident in a free market western country such as the U.S. More important, the enterprise plays a double-role economically, by both generating the demand for services and by providing the supply of services to satisfy this demand. The demand is generated through setting of medical standards, like the therapeutic thresholds for common medical conditions; through the determination of the most appropriate treatments and medications for medical needs, as done by the physicians and the drug companies; and, mostly, by being the only entity that determines the health status of individuals and thus their medical needs. In this regard, professional societies have a major role, as they essentially "capture" the guidelines process; of course, they also have conflicts of interests. Kaplan and Ong[23], for example, suggest that the medical enterprise has effectively expanded their market by creating guidelines suggesting that 97% of the adult population would need to be under medical surveillance. The enterprise has incentives and resources to keep their market share healthy.

The healthcare enterprise is the one determining how medically needy individuals will be treated. Clearly, this is a situation where the "cat is asked to safeguard the cream", where the enterprise would first of all maximize its profits, while assuring that it is legally protected against malpractice liability. The government, in its role as the guide of the financial market, promotes this attitude. Consequently, the enterprise tends to replace less profitable treatments and services with more profitable ones, use certain services excessively (e.g., as is the case with hysterectomy and caesarean operations), add many diagnostic services to protect against liability, or over-perform diagnostic services just to protect itself from malpractice suits. The powerful healthcare enterprise has essentially no incentive to promote upstream prevention efforts, or to divert resources to non-medical approaches to enhance population health.

The healthcare enterprise also has the incentive and extensive financial resources to perpetuate the demand for its products and services, regardless of their impact on the long-term population health. Just consider how the amount and length of time needed for the development of a new drug creates barriers to entry for new entities to the drug development and manufacturing business. The result is the continuity and power of the existing pharmaceutical industry. At the same time, these resources support an extensive lobbying activity that protects the interests of the drug companies, and ensure that medical providers use their drugs; both activities further strengthen this sector. Similar observations can be made with regard to the large healthcare organizations, the insurance companies, the societies of medical professionals, and the other members of the healthcare enterprise. Although enterprise may advocate for competition, they also seek to control competition, as evidenced by the assertive campaign against a public option in U.S. healthcare reform.

One of the most significant problems for individual consumers is the manipulation of their preferences by direct to consumer advertising. Although the advertising is alleged to provide consumer information, substantial evidence suggests that these advertisements offer limited information, are beyond the comprehension of many consumers, and are designed to empower consumers to request specific products by brand name. The result is overselling of products that add to the excessive misuse of the healthcare system, promotes and strengthen the enterprise, and might conflict with the goal of promoting population health[24].

The government also contributes to the imbalance of the market. Like the healthcare enterprise, the government has considerable amount of resources and information - far beyond the resources and information available to the individuals and the public. Through directed and targeted release of this information, it affects public expectations and legislative and control activities. Using reimbursement to enterprise providers for medical services, as in the UK's NHS or the U.S.'s Medicare system it maintains the status quo in the market and assures continuance of current practices. The government has, in addition to vast resources, considerable legislative and control power. Through enterprise-like activity, such as Pay for Performance in the UK and Fee for Service as employed by the CMS in the U.S., it influences the demand and supply in the market. At the same time, due to the limitations of the political process as were evident in the dialog pursuant in the U.S. healthcare reform, government decisions often are made with insufficient pertinent information; this was also evidenced by some of the latest incidents regarding certifications of medications by formulary control authorities.

Allowing government to directly compete with the enterprise as a provider of health services and payer of health insurance, may further upset the balance in the market, threaten enterprise providers, and reshape the market. The introduction of the Massachusetts health care reform law in 2006, which mandated that nearly every resident of Massachusetts obtain a state-government-regulated minimum level of healthcare insurance coverage, completely changed the healthcare market in Massachusetts. In Israel, the introduction of the National Health Act at the end of 1994, and the resulting effective nationalization of the four Israeli sick funds, created a government controlled healthcare system, that only in the last several years have been penetrated in a significant share by the insurance companies[25]. The level of government involvement was, and continues to be, one of the major contended issues in the U.S. healthcare reform that was enacted earlier this year.

### Consequences of the Imbalance

The consequences of this unhealthy imbalance between the three actors include excess use of unnecessary services and underuse of some valuable services. We believe that these are a direct consequence of the imbalance between enterprise and government, and the weakness of the individual and the public; this is well demonstrated by the move toward overtreatment of blood pressure and cholesterol as discussed below. Yet, healthcare reform in the U.S. will do little to control this influence of enterprise.

### Excess

Health services, as argued above, should improve population health by extending life and improving quality of life for the entire population. Excess services are activities that use resources without enhancing population health, or services inappropriate for health needs. Excess health services are closely related to disproportionate distribution and use of healthcare resources. Those with easy access to services tend to overuse them, or have excessive services and medications offered to them by providers and drug companies. On the other hand, those with poor or missing health insurance tend to minimize use of preventive and ambulatory day-to-day resources, and excessively use publicly available, higher cost services, such as emergency rooms at time of stress.

**Figure 3:**
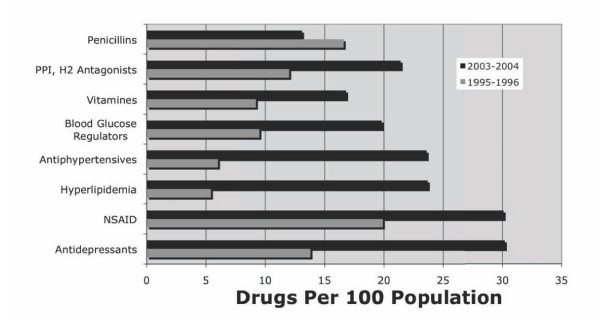
**Change in Use of Selected Prescription and Non-Prescription Drugs between 1995-6 and 2003-4**. Data from Health, United States 2006 Data from table 92, pg. 332 (art original)

Demands on resources continue to grow. Aging and chronic illnesses may be associated with a disproportionate share in the growth of healthcare costs. A series of papers by Thorpe illustrate this point[26,27]. For example, 75% of total healthcare spending in the US is linked to chronically ill patients. Over the last twenty years there have been substantial increases in the proportion of the population treated for chronic conditions. For example, the proportion of the population being actively treated for high cholesterol jumped from 1.4% of the population in 1987 to 10.7% in 2003 (an increase of 664%). The number of adults treated for mental disorders nearly tripled in this interval from 5.3% to 17.4%[26]. Adjusted for inflation, the U.S. spends less on hospital care than it did twenty years ago, but more than twice as much for prescription drugs[23]. Figure 3 shows the changes is the use of common drugs over a 10 year period beginning in 1995-1996 and ending in 2003-2004. The data are based on visits to physicians that are coded as drug visits. These are defined as outpatient office or hospital outpatient department visits during which at least one prescription or non-prescription medication was recorded in the patient record. They are presented in two-year blocks: 1995-1996 and 2003-2004. The raw numbers were divided by the sum of the population estimates for both years in each block, and divided by 100 to produce the number of drugs per 100 persons in the population.

Treatment of chronic illness has been attractive to enterprise actors: medical providers and pharmaceutical companies. Those with chronic disease have an ongoing need for medications and for the regular use of medical care. For most of these conditions, the cost of medication use significantly exceeds the cost of hospitalization[27]. Between 1995-6 and 2003-4, prescriptions for penicillin decreased by 21 percent. However, antidepressant medication prescriptions increased by 118%, antiphypertensive prescriptions increased by 293% and drugs for high cholesterol increased by 339%! As those born after the second World War reach retirement age with their chronic illnesses, the percent of total healthcare costs spent on prescription medications can be expected to increase[28], with a resulting increase in the overall healthcare costs.

### Enterprise Forces that Expand Care

One of the most important mechanisms for promoting the goals of the enterprise is through the use of evidence-based guidelines, which have a substantial influence of the services that will be delivered. Though supposedly founded on evidence-based reviews, the guidelines often lead to inefficient use of resources. Further, there are many different groups creating guidelines; unfortunately, conflicts of interest in guideline committees remain common[29].

Peer developed guidelines legitimize much of contemporary healthcare. Treatment for the very high prevalence of chronic disease accounts for as much as two thirds of healthcare spending[28]. It is not entirely clear that this level of expenditure is appropriate. Over the last few years, the diagnostic thresholds for several common medical conditions have been lowered, resulting in a substantial expansion in the market for healthcare[30,31]. For example, the majority of the older adult population now meets the criteria for hypercholesterolemia and hypertensive disease as defined by the most recent guidelines for high cholesterol[32] and high blood pressure[33], and are, consequently, in need of regular medical attention. In the last few years, proposals have emerged to lower disease thresholds for these and other conditions even further.

Consider blood pressure, for example. The number of people affected would go from about 14% to about 40% of the adult U.S. population if the new guidelines are adopted. In other words, there would be a 185% increase in the number of affected people with no observable change in the status of the population[23]. The Joint National Committee on Prevention, Detection, Evaluation, and Treatment of High Blood Pressure, in its JNC-7 report, created a new category known as pre-hypertension; individuals previously categorized as normal now qualify for this diagnosis[34]. The National Cholesterol Education Program Adult Treatment Panel III Guidelines (ATP-3) lowered the threshold for concern about serum cholesterol[35]. A committee of the American Diabetes Association lowered the threshold for Impaired Fasting Glucose from 110 mg/dl to 100 mg/dl[36]. Analysis of the National Health and Nutrition Examination survey shows that the change of definitions has profound effects on the number of people who might be labeled as having these three risk factors: cholesterol, blood pressure, and blood glucose. Kaplan and Ong[23] explored the implications of changing the definitions of Coronary Heart Disease (CHD) risk factors. Considering these three risk factors, more than 97% of the adult population would need to be under medical surveillance. Kaplan and Ong [23] suggest that the benefit of treatment for adults in the "pre-disease" categories may be quite small. On the other hand, the costs of the treatment are likely to be substantial. New pharmacological approaches to the treatment of high blood pressure are very expensive, costing several dollars per day. For a 50 year old diagnosed with "pre-hypertension," the additional drug costs might exceed $500 per year, and this cost would be repeated each year for the remainder of that individual's lifespan. In addition, there are substantial monitoring costs because people taking medications need to visit their physicians more often.

It is worth noting that different groups that look at the same data may decide on different guidelines. For example, the European Society of Hypertension Task Force recently backed away from the aggressive management of low threshold hypertension, blood glucose, and serum cholesterol [37,38].

### Under Use of Potentially Valuable Services

At the same time that ineffective services are being overused, many potentially valuable services are not used enough. For example, systematic evidence suggests that at the time of admission to the hospital for a heart attack, and at the time of discharge, the administration of beta blockers and aspirin can save lives. But, these inexpensive and effective interventions are underutilized[39].

Individuals also play a role in the overuse of healthcare, because of their motivation to stay well. People who have higher anxiety about their own health and insufficient information, may develop an affinity for treatments, and especially if encouraged to do so through drug promotion and advertising, as is common in the U.S. Enterprise may have undue influence on information and may control markets through the development of guidelines and through advertising. Government may yield to the persuasive position of the enterprise and its political power. At the same time, many principles that could have an important impact on community health are overlooked, largely because they do not have the right advocates.

Prevention is perhaps the best example reflecting misalignment of incentives. There may be many ways to prevent disease, but modern preventive medicine favors screening tests such as mammography or the Prostate Specific Antigen (PSA) test. However, screening is secondary rather than primary prevention, because it detects a disease that has already been initiated. Much of what we believe to be "early" detection is really detection of disease at a relatively advanced phase. Randomized clinical trials suggest that the impact of cancer screening on total mortality is quiet disappointing. Although it seems counterintuitive, virtually all systematic randomized clinical trials that consider total mortality fail to show that cancer screening extends the life expectancy[40].

In 1993 McGinnis and Foege forced a reevaluation of health risks by concentrating on the major non-genetic contributors to mortality[41]. When these factors are considered independent of the disease model, clear priorities for prevention emerge. Tobacco use is associated with more than 400,000 deaths each year, while diet and physical activity patterns account for an additional 300,000. These dwarf the number of deaths associated with problems that the public is generally concerned about, such as illicit drug use. The McGinnis and Foege analysis was replicated in the year 2000 by Mokdad and colleagues[42]. The results of their analysis are shown in Table 1. The updated analysis revealed the same rank ordering of the actual causes of death. However, poor diet and physical inactivity were gaining on tobacco use as the leading actual cause of death.

In the previous section we cited several examples of commonly used interventions that can be expected to have minimal effects on health outcomes. Conversely, other services that might have substantial population impact are often less used. Enterprise, which is highly invested in heath insurance coverage, focuses on treatment for defined diagnoses. Typically, only a tiny portion of the huge healthcare budget is devoted to interventions aimed at the main causes of sickness and mortality, most of which are behavioral. Estimates suggest that less than 5% of the total annual healthcare budget is devoted to prevention efforts (Rothenberg, Masca, Mikl et al., 1987). Nearly all of this 5% is used for secondary prevention, such as cancer screening tests.

Coffield and colleagues[43] reviewed the cost/effectiveness of preventive services evaluated by the U.S. preventive services taskforce. Their analysis considered two dimensions: the amount of disease burden relieved by the service, and the cost/effectiveness of the intervention. The highest impact service with the lowest rate of delivery was tobacco cessation interventions for adults. Less than 50% of adults who could benefit from this intervention actually receive it. Other underutilized effective services were screening for undetected vision impairments, and substance (including tobacco) prevention programs for adolescents. In order to determine how best to use scarce healthcare resources, we need to analyze the benefits and outcomes of health related interventions in relation to the resources they require[44]. Enterprise has little interest in promoting these potentially effective services, and individuals lack the understanding of how they can effectively enact behavior change. Government, although clearly supportive of primary prevention, lacks the public health infrastructure or the incentive and resources to successfully implement primary prevention programs.

### The Healthcare Pareto Efficiency

**Figure 4:**
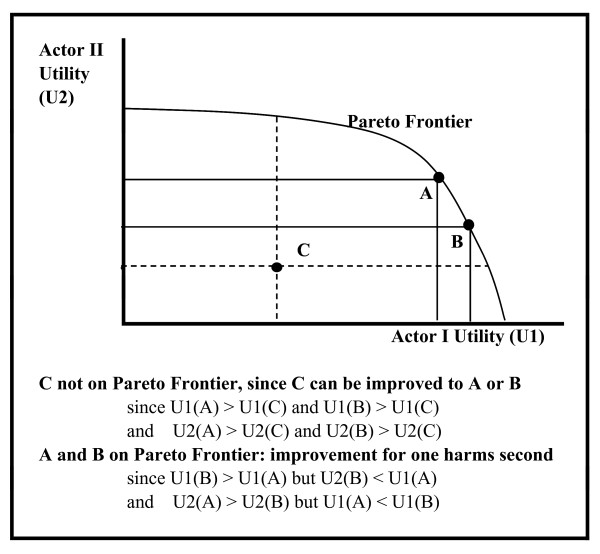
**Exanmple of Pareto frontier**.

How does the economic balance, or rather the imbalance, between the three groups of actors lead to the healthcare market inefficiency? Can this balance be shifted to reduce or eliminate the opportunity cost dilemma? To this end, we consider whether the market is Pareto efficient or optimal[45], using the concept of Pareto frontier (Figure 4); this will be later used to analyze the U.S. healthcare arena.

Each actor has a set of resources that he can allocate according to his utility. These include supplies such as drugs and hospital beds; activities such as treatments and training; resources for improved health such as physician visits and lab tests; or time and money that a patient can allocate to health related activities. The resources need not be all monetary or monetary equivalent; information, for example, is a hard to quantify resource, yet it substantially affects the outcomes of the system. The actors allocate their resources to achieve a joint outcome. The utility of each actor determines the value of the outcome to this actor, leading to a preference order of allocations for the actor; the higher the utility, the more preferred the outcome. Different actors may have different utility functions, and thus different preferred order for the outcomes. For example: the patient may place a higher value on his health, and particularly with regard to severe ailments, while the drug company may value more its market share or its profits.

A particular allocation of resources by an actor reflects his utility and use of resources at the time of allocation. At each point in time the set of allocations by all the actors determines the position of the market and the outcome for each actor. When a move from one allocation to another can improve the outcome for at least one actor, without harming other actors, it is a Pareto improvement. When the outcome of an allocation cannot be improved by any Pareto improvement allocations, it is a Pareto efficient allocation. The set of all Pareto efficient allocations is the Pareto frontier. Point A in Figure 4 demonstrates a position on the Pareto frontier for actor II, for example, the position of the drug companies in the healthcare system. Note that while a system may be Pareto efficient, it may function in a "local optimum"; i.e., there may be another Pareto efficient allocation which may be preferable, at least for some of the actors (as demonstrated by point B in Figure 4), but it may not be reachable through (local) Pareto improvement allocation movements. It is also worth noting that Pareto efficiency does not require an equitable distribution of wealth; the system may be Pareto efficient even if a small minority has the vast majority of the resources (as is the case with the drug companies in the U.S.), or benefits from a much higher level of its utility than the other actors.

One should distinguish between the utilities of the individual actors, and the "combined utility" of the whole market. The combined utility is a weighted combination of the individual utilities, but its optimal value (for the whole market) may be reached in a position that does not maximize the utilities of one or more of the actors. The allocation of the healthcare system to achieve the highest population health status and outcomes, for example, may result in reduced profits (at least for the short run) of some of the health enterprises.

## Discussion

If a system is not on the Pareto frontier, then it is not Pareto efficient and thus, in principle at least, there is a potential for Pareto improvement. For example: in Figure 4, point C is not on the Pareto frontier, as both actors may gain (or at least not experience a decrease in their utility) by moving to points A or B, or to any point on the Pareto frontier between A and B. In reality, though, even such a Pareto improvement may require compensation or forced choice for one or more of the actors; this may be due to the aversion to change of many organizations, and to the short-term costs of a change.

When the system is on the Pareto frontier, the actors - as a group - have no incentive to deviate from the status quo, and the resulting problems or inequities currently existing in the market. Indeed, a system wherein strong actors take goods from the weak, as is the case with the healthcare U.S. system, may be Pareto efficient[46]. In other words: the current healthcare arena may be riddled with issues and problems, but it is still hard to change because it remains in a (relatively) stable position. The outcomes of the U.S. health reform demonstrate this; the relative positions of the public, enterprise, and government, and the mechanisms governing the functions of the market, may not substantially change as a result of the reform. Furthermore, it can be argued that it is essentially impossible to generate, with the existing actors and preferences, a globally accepted utility, which aggregates the preferences of the various actors into market preferences, while keeping their preference order, so as to achieve a market-wide acceptable and stable position^2^.

At the same time, as noted above, change over the Pareto frontier can be achieved, but only at a cost to one or more actors. For example, the U.S. healthcare reform offers the opportunity for basic healthcare insurance to many who are currently uninsured, and thus reduce the high costs that result from their current use of emergency services. But this will reduce the hospitals income from emergency service (e.g., through Medicare Disproportionate Share (DSH) payments and other reclaiming programs). Consequently, only the passage of a major legislation, such as the U.S. Patient Protection and Affordable Care Act, could force such a move. Note, however, that this will result in a larger efficiency of the system, increased numbers of insured (and thus premium paying) customers, and reduced emergency services costs; thus, it is expected to improve the overall economy of the healthcare system and its participants.

**Figure 5:**
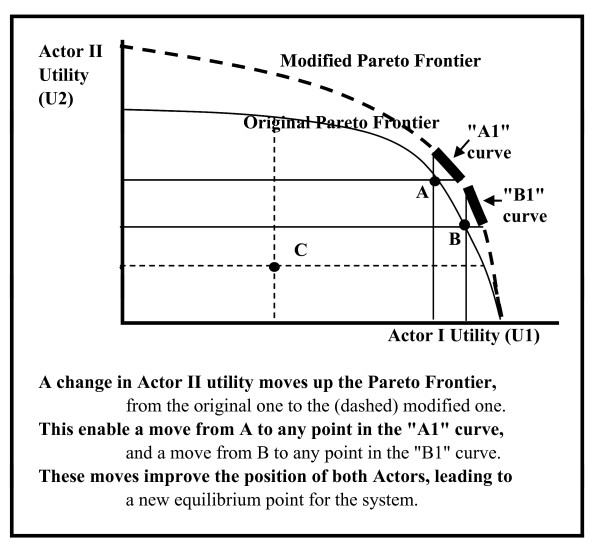
Example of change in Utilities

Another mechanism for a change in the allocations is a change in the utility functions of the actors. Such a change eventually leads to a new Pareto frontier, thus enabling Pareto movement to the new frontier. For example: a change in the utility of Actor II in Figure 5 shifts the original Pareto frontier upward into the modified Pareto frontier. This shift provides opportunity of a shift from the "A" and "B" equilibrium points on the original Pareto frontier to any point on the "A1" to "B1" curved sections on the modified Pareto frontier. Because much of the positioning of the actors in the healthcare market is due to public expectations, change in these expectations may lead to changes in the utilities and thus to a change in the Pareto frontier. The change in the attitude toward obesity, for example, is responsible for major shifts in the positioning of various treatments, diets, medications and behaviors.

Certain actors have more power to affect the market. The medical profession affects the demand for medical and health-related services, through the rejection of certain treatments or by a change in the thresholds for treating disease. The enterprise affects the market through advertising and products promotions. The government affects the market through legislation, certification, customer education, modified Medicare and Medicaid payment schedules, and changes in supporting services such a mental health treatment.

The healthcare system is characterized by a short-term perspective. The government has essentially an election-to-election planning horizon, and the enterprises are mostly driven by short-term financial and profit objective. A longer-term enterprise perspective should consider healthcare strategy and investment to be a Socially Responsible Investment^3^, especially as related to the social, ethical, and corporate governance. In the U.S. the Health Insurance Portability and Accountability Act of 1996 (HIPAA) created, with a long-term perspective, strict rules for the protection of individually identifiable health information. HIPAA took major steps toward social responsibility by recognizing the rights of patients and forcing significant changes in the enterprise procedures, data management, and activities. The Sarbane-Oxley Act, created by the U.S. Congress in 2002, was deigned to protect investors from corporate accounting fraud. This helped boost corporate responsibility and transparency. Standards such as International Financial Reporting Standards (IFRS), Basel II and Solvency II, and their related regulations, considerably change the long-term accounting and business environment, with the objective of establishing more stable, secure and transparent markets. If the major government and enterprise players will adopt a long-term healthcare perspective, they may recognize that improving population health is, in the long-run, a winning strategy that can pay back more than the short-term profit maximization approach currently used.

This brings us back to our premise - that healthcare resources should buy the most health for the most people. This is, in effect, the individual and public actor long-term utility, though not the apparent enterprise actor utility. We argue that if the government is indeed to fulfill its constitutional responsibility as the public advocate and provide for the general societal welfare, it should adopt the same utility and a long-term perspective. The government has both the information required for this task, as well as the legislative and financial means to act. In principle, the government recognizes this responsibility; however, the interpretation of "general welfare" is fraught with ideological and political overtones, and particularly so in the U.S. The starkest example is the issue of a national health program that provides services to all residents. Many countries, such as the U.K. and other western countries, have adopted national health systems, or a combination of a national health system in cooperation with private market enterprises. The U.S., on the other hand, values a capitalistic system wherein the means of production and services are mostly privately owned and operated for profit, and the economic means are distributed through the operation of a market economy; these, of course, contradict a health system in which a central authority determines the distribution of healthcare resources and outcomes. In this U.S. system, societal objectives tend to be ignored by the enterprise which provides the services, and thus without governmental intervention the objective of buying most health for most people is ignored. Indeed, it took more than four decades to achieve even a modest health reform in the U.S., and only one state (Massachusetts) has a healthcare system that resembles a "national" health environment.

The very fact of a relatively stable U.S. healthcare market implies a Pareto efficient status, and thus little prospect for an un-enforced change. Indeed, it required a major political drive by President Obama and the Democratic Party, fuelled by strong public demand, for a health reform to be passed in the U.S. in 2009-2010, which modifies various aspects of the existing U.S. healthcare system The new reform package will create insurance exchanges that increase consumer purchasing power, and tax incentives for employers to offer insurance. The program will forbid the denial of insurance to those with pre-existing health conditions, and will mandate the purchase of insurance by most citizens. Consequently, government will play a stronger role in regulating the interface between enterprise and individuals.

## Summary

Due to the relative weakness of the individual/public actor, and the strong position of the government in its role as the representative of the public, it is clear that major changes will occur only when the individual and public actors will work together. We believe these two actors can become better aligned, as was partially done under the U.S. Patient Protection Affordable Care Act. To strengthen the individual/public sector, there is a need for improved information. A clear campaign is required to improve access to information, improve transparency, educate the public, provide reliable sources of information, regulate the extent and accuracy of advertising claims, and respond to questions and requests for information. This may require an information revolution similar to the privacy and patient rights revolution of the last decade. One step in this direction is the rapid deployment of a global Medical Health Record, which will enable sharing of information when needed between all the health actors. The record will improve the knowledge available to healthcare providers, and thus prevent errors, cut costs of duplicate services and excessive testing, and support better healthcare services. At the same time, it may expose personal medical information to a wider audience, and thus is fraught with many social and legal issues.

Greater involvement of patients in decision-making should be a priority. Patients make good decisions when they have good information. Often this requires clearer information about the risks of treatments and the recognition that foregoing treatment may be an option. To help patients become more involved in decision making about their own care, the use of shared medical decision-making and decision aides should be expanded[47-49], and programs such as Medicare and Medicaid should pay providers for the extra time required to deliver these services.

The healthcare enterprise offers medical and surgical treatments that can have dramatic benefits [15]. However, there are cases in which treatments have relatively little effect on health outcomes and may consume resources without offering sufficient benefit[50]. When too many dollars are used in pursuit of treatments with lower value, less is available to purchase services that are truly necessary. Sometimes overuse of services may be stimulated by direct-to-consumer advertising campaigns. Direct-to-consumer advertising of pharmaceutical products is only allowed in two countries, the United States and New Zealand, and there is legal precedent for control over this "commercial speech". Federal agencies should require balanced presentations of costs, risks, and benefits, in a language and format understandable by the average consumer.

The information available to decision makers, the government, and the public must improve. While technology enables us to access enormous amount of data today, restrictions of privacy, data ownership, and (often antiquated) laws and traditions prevent efficient use of this data, and its conversion to useful information. We are also lacking objective experts and models that can effectively use this data and distribute the resulting information. Some action steps that should be considered include:

• Improve and enhance the transparency requirements for all parties, whether private or governmental. In addition to full disclosure, the information should be presented in a way that can be understood and absorbed by the public.

• Evidence-based guidelines developed by impartial groups should be required to consider both costs and opportunity costs. An impartial governmental agency should regulate the guideline process.

• Although conflicts of interest are now disclosed for most independently promulgated guidelines, the analyses should report who is likely to benefit and who will bear the costs, as well as the relations of the guidelines setters with those who will benefit.

• Guidelines should improve transparency by reporting both the absolute and the relative risk outcomes for people at varying levels on initial risk. Compliance with the guidelines will affect the likely increase in market size for pharmaceutical products and healthcare services.

The government and public agencies must lead these efforts; this, of course, requires political will and continuous public pressure. Although difficult, we believe these goals are attainable.

### Limitations

It is important to recognize that healthcare decisions are the product of a wide variety of influences including cultural, historical, political, economical, legal and professional factors. The analysis offered here is simplified to highlight and draw the contrast between just three major actors, and describe the forces that shape the current equilibrium. There are also important influences at the public policy, social environmental, and the individual decision maker levels. Thus we encourage further analysis that builds more complex models and considers the many nuances that will be required to advance policy shaping and change.

## Abbreviations

(CMS): Center for Medicare and Medicaid Services; (CHD): Coronary Heart Disease; (GDP): Gross Domestic Product; (JNC): Joint National Committee on Prevention, Detection, Evaluation, and Treatment of High Blood Pressure; (ATP-3): National Cholesterol Education Program Adult Treatment Panel III Guidelines; (NHS): National Health Service; (PSA): Prostate Specific Antigen; (U.S.): United State; (WHO): World Health Organization

## Competing interests

This work was completed while the first author was supported by grants from the US National Institutes of Health, the Agency for Healthcare Research and Quality, and the Centers for Disease Control. We did not receive support from any parties with direct financial interest in the conclusions of this work.

## Authors' contributions

The authors were equal partners in the development of the manuscript.

## Endnotes

^1 ^As noted by one of the reviewers, there is a sufficient evidence of the weaknesses of QALY in capturing important aspects of value, and that - as well as the lack of consensus over goals - led to the development of process-based decision-making frameworks. We do not promote QALY as the sole, or prominent, measure; rather, we use it to demonstrate that such health measures exist.

^2 ^The situation in the healthcare market can be interpreted as a "voting" for resources. For voting systems, it is said that "the only non-flawed voting system is a dictatorship" (in the healthcare arena - by the enterprise and government actors), as a result of Arrow's Impossibility Theorem. See, e.g., Why Flip a Coin? The Art and Science of Good Decisions by H.W. Lewis, Wiley, 1997, ISBN 0-471-29645-7.

^3 ^See. E.g., the European Social Investment Forum, (http://www.eurosif.org) or the Social Investment Forum (http://www.socialinvest.org), and their publications.

## Pre-publication history

The pre-publication history for this paper can be accessed here:

http://www.biomedcentral.com/1472-6963/11/85/prepub
